# Student motivation to study music and sport – a comparison between study subjects and study programs on intrinsic and extrinsic motivational aspects

**DOI:** 10.3389/fpsyg.2024.1393339

**Published:** 2024-08-13

**Authors:** Anna Immerz, Manfred Nusseck, Jesper Hohagen, Claudia Spahn

**Affiliations:** Freiburg Institute of Musicians’ Medicine, University of Music Freiburg, Freiburg University Medical Center, Faculty of Medicine, University of Freiburg, Freiburg Center for Music Research and Teaching, Freiburg, Germany

**Keywords:** intrinsic motivation, extrinsic motivation, motivation for enrollment, university students, music, sport

## Abstract

**Instruction:**

In both subjects, music and sport, the engagement with the subject content – learning an instrument or training in a sports club – usually begins in early childhood. This makes these subjects special and similar. It is therefore of interest to examine the motivation for choosing music and sport as subjects for university study.

**Methods:**

In the present investigation, 151 students at the beginning of their university studies were examined. Among these were 110 music university students in the Bachelor of Music (B.Mus.) and music teacher education program, and 41 sport university students in the Bachelor of Science (B.Sc.) and sport teacher education program. The study contained a custom-made questionnaire on sociodemographic data, questions on study choice alternatives and biographical musical or sporting background, as well as two standardized questionnaires on motivation for enrollment (STUWA) and on aspects that are important for the profession.

**Results:**

Music and sport students were at a rather high and similar level of intrinsic motivation to study their subjects. However, materialistic goals motivated the teacher education students more than the bachelor’s students with an artistic program. The difference between the study programs was also found in the context of extrinsic-social motivation, where the teacher training students answered with higher scores. With regard to socially-induced motivation, it was shown that music students and sport teacher education students were more socially induced to study the respective subject compared to the general student population. With regard to uncertainty when choosing a course, it was found that Bachelor of Music students were more certain that they wanted to study exactly their particular subject. The ages at which music and sport were started in childhood were similar, but the first-year music students were younger than the sport students were. Compared to sports students, music students decided their area of study earlier, and bachelor’s students in music in particular had fewer alternative study options than sports students.

**Discussion:**

The results provide a differentiated picture of student motivation to study and thus allow a deeper insight into the subject cultures of music and sport. They also open up opportunities for follow-up studies in comparison with other study subjects and programs.

## 1 Introduction

### 1.1 Clarification of the terms motivation and study motivation

The term motivation originates mainly from the psychology of learning and motivation and is often used in the context of learning motivation (Myers and DeWall, 2023). Motivational and learning psychology asks what drives a person, where this drive comes from, what a person wants to achieve with their behavior and what it is aimed at (Bak, 2019). Reasons for motivation can be differentiated between intrinsic and extrinsic motivation. *Intrinsic motivation* is the desire to perform a behavior for its own sake. *Extrinsic motivation*, on the other hand, describes the desire to perform a certain behavior because of expected rewards or the threat of punishment (Myers and DeWall, 2023). While intrinsic motivation thus arises from a person’s inner desires and needs, external motivation is initiated by environmental factors and relevant reference persons and groups. The differentiation between intrinsic and extrinsic motivational factors plays a central role in the discussion about university students’ motivation for study in higher education.

According to Großmann (2016), many research studies analyzing study motivation use this term without precisely defining the underlying construct and its components. Motivation is understood and used in different ways in relation to the choice of a university subject. In German-speaking discourse, the terms *study motivation* [Studienmotivation], *study choice motivation* [Studienwahlmotivation] or *career choice motivation* [Berufswahlmotivation] are used quite synonymously. In the English-language literature, on the other hand, the focus tends to be on careers with terms such as *career decision* and *career choice*.

In his reflections on study motivation, Großmann (2012) assumed that students pursue certain goals and interests with their studies. He defines study motivation as a “set of specific attitudes and expectations that are linked to completing a university degree” (Großmann, 2012, p. 447).

In this publication, we use the term *study motivation* as a general concept to describe the motivation of university students (see also Grüneberg and Süß, 2023). Following Großmann (2016), we understand study motivation as a hypothetical construct that explains the type of action and behavior in the context of studying. In this sense, it means the individual attitude toward studying as well as the decision to study in general, the motivation for enrollment and the decision for a particular subject or study program at a particular university (Großmann, 2016).

### 1.2 Study motivation in higher education research

Study motivation is currently being discussed in several fields of higher education research. On the one hand, study motivation plays a prominent role with regard to changing subjects or student drop-out from higher education institutions (Heublein, 2014; Heublein and Schmelzer, 2018; Messerer et al., 2023). In addition, the relation between motivation and academic success (Heinze, 2018; Hillebrecht, 2019; Baalmann and Speck, 2020; Leichner et al., 2022; Stellmacher and Paetsch, 2023) or the link between study motivation and study satisfaction (Künsting and Lipowsky, 2011; Kaub et al., 2012) or teacher health (Schüle et al., 2014; Janke, 2020; Merkle et al., 2023) has been investigated in numerous studies. Also, study motivation subsumes individual orientations and motivations for starting a university degree (Bornhorst et al., 2020) or choosing a particular subject (Piroth, 2013). On this basis, Großmann (2016) emphasizes that study motivation is an important model for researching subject cultures [*Fachkulturen*] – such as music or sport.

Subject-specific differences are evident in a survey among university students in Germany on their values, goals and perspectives (Hinz, 2022). The students were asked about their motives for choosing their study subject, revealing a gender-specific difference: compared to women, men attached more importance to good earning potential (men 41% vs. women 32%) and career opportunities (men 40% vs. women 31%) when choosing a degree course. Women, on the other hand, attached much more importance to personal interest (women 59% vs. men 50%) as a motive for choosing a subject than male students. There were also differences between the study subjects. When choosing a course of study, personal interest was particularly frequent as a major reason among university students of the humanities (78%), cultural (75%), social (67%), natural (59%) and linguistic/literary sciences (54%) as well as among university students of medicine (61%). Only for law (51%), engineering/computer science (48%) and economics students (44%) earning opportunities were the more important motives. Among the humanities students, for example, only 24% stated that good earning opportunities were a very important reason for their choice of degree course. No conclusions were drawn from the results of the study about music and sports university students.

In a qualitative study on career orientation patterns and the study motivation of bachelor university students from different study programs and subjects, Bornhorst et al. (2020) found a high level of intrinsic study motivation with a high level of commitment to the subjects over all university students. It became clear that biographically based career goals – based on experience, internships, etc. – were already formed before the start of the degree course and were specifically implemented by the students during their studies. The authors (Bornhorst et al., 2020) also emphasize that the issues surrounding the topic of study motivation are highly complex and that the educational biography, the identity formation process of the students and their social background should also be taken into account. Janke et al. (2023) pursued a quantitative approach to recording study motivation and therefore developed a reliable and valid instrument to measure different facets of study motivation and motivation for enrollment (STUWA: Ein multifaktorielles Inventar zur Erfassung von Studienwahlmotivation, Janke et al., 2023). Conventional inventories often focus on specific study programs (e.g., FEMOLA: Fragebogen zur Erfassung der Motivation für die Wahl des Lehramtsstudiums; [Questionnaire to assess motivation for choosing a teaching degree program] Pohlmann and Möller, 2010). The STUWA questionnaire, on the other hand, is a generalized multifactorial scale for recording study motivation that can be used in all subjects and in various study programs. This enables group comparisons to be made between students of different subjects with regard to their study motivation. Thus, Janke et al. (2023) found interesting results in group comparisons between business students and student teachers: Teacher education students reported an extrinsic-social and socially induced study motivation, while business students in comparison to other students reported higher extrinsic-materialistic motivation for enrollment. Student teachers decided to study teacher education and to become a teacher because they were encouraged by friends, family, or colleagues (socially induced motivation). The compatibility of family and career was also important to them when making their decision. They want to have time for family, friends, and hobbies in addition to their career (extrinsic-social motivation). Business students, on the other hand, showed that extrinsic-materialistic aspects such as a good income later on and financial security seemed to be more important to them.

### 1.3 Study motivation of teacher education students

As mentioned above, teaching is a well-researched profession and there are a large number of studies on the motivation of prospective teachers and their decision to study to become a teacher (Pohlmann and Möller, 2010; Rothland, 2014; Besa, 2018; Grüneberg and Süß, 2023; Janke et al., 2023). As an example, the publication by Grüneberg and Süß (2023) is examined in more detail. The authors found that an intrinsic study motivation was the main reason of first-semester university students to choose a teacher education program. The university students want especially to support children and young people in their personal development and education. Thus, most student teachers are united by a high level of professional interest. The authors also compared the results of intrinsic motivation in relation to the type of school, showing that a strong subject orientation prevails among prospective secondary school teachers. On the other hand, prospective primary school teachers are more interested in the pedagogical-educational challenge of working with students at school. These results can also be seen in the study by Retelsdorf and Möller (2012), where they analyze the association between the education study program – primary and secondary school – and the motivation for choosing teacher education. They observed that subject interest was strongly related to choosing a teacher program for secondary school, whereas educational interest was rather related to prospective teachers in elementary school. As a further result, Grüneberg and Süß (2023) found that student teachers tended to agree with the statements that the teaching profession offers good employment opportunities, flexibility, free time, and compatibility with family life. Against this background, the majority of students in the study stated that they were quite sure about their decision to study teacher education. This is also reflected in the answer to the question about alternatives: teacher education is the preferred choice of study for 87.8% of the students asked.

In addition to the quantitative part of their study, Grüneberg and Süß (2023) also conducted interviews with student teachers about their study motivation. When asked about the main reasons for their choice of study and their motivation to study, the participants primarily responded with professional status and ideal career “teacher” as well as subject interest, study content, and exchange of content. They also cite practical teaching experience during their studies as a further reason for motivation.

Only a few studies in teacher education deal with study motivation in individual subject cultures, such as Piroth (2013) with the study motivation and career expectations of students in Protestant religious and community education, or Fischer et al. (2019), who compare sport and mathematics students. As part of our study, we were interested in how motivation to study behaves in specific subject cultures such as music and sport. Previous studies on the fields of sport and music are therefore described below.

## 2 Subject-specific perspectives on music and sport

### 2.1 Studying sport

Sport students bring with them a wide range of biographical experiences in sports – from school to extracurricular activities. Sports socialization, which is often linked to sport clubs, usually begins very early, sometimes as early as preschool age (Lüsebrink et al., 2014; Wyllemann et al., 2020). Klinge (2002) thus speaks of an athlete or a sport habitus acquired prior to studying. In his model of the professional biographical development of sport teachers, Miethling (2018) attributes the development of sporting lifestyles to childhood and adolescence. In this phase, habitual patterns are acquired through milieu-specific sporting and educational experiences.

Wylleman (2019) illustrated the career of athletes in his holistic athletic career (HAC) model, defining six stages of development, each stage consisting of different successive phases. Thus, the model reflects the development of athletes’ careers on a sporting, psychological, psychosocial, academic/professional, financial, and legal level of development. The discourse on the decision to study sport takes place at the point from phase two to phase three – the athletic level between development to mastery stage. Wylleman (2019) gives an approximate age of between 18 and 20 years for this psychosocial transition between adolescence and young adulthood in his model. On an academic and vocational level, the transition is accompanied by a change from school to university and thus from secondary to higher education. Peers, coaches, parents, teammates, and sports students are important in this process on a psychosocial level.

While Wylleman (2019) approaches the biography of athletes on a conceptual level, Volkmann (2008) chooses an empirical approach in her work on the biographical knowledge of teachers. She examines the influence of biographical experiences in the subject of sport. With her qualitative analysis, she was able to show that biographical knowledge has a major influence on the professionalization process of sport teachers and its progression. She reconstructed from her data that personal sporting activities and enthusiasm for sport are of great importance for the career choice. The practical sports components of the degree course are just as important for teachers working in the profession (Volkmann, 2008).

In their study, Streblow and Brandhorst (2018) looked at prospective teachers in sport and were able to differentiate between three types of students based on their motives for choosing teacher education. When comparing the three types, Streblow and Brandhorst (2018) found no difference in study motivation between sport students and non-sport students. However, they found a significant correlation between the study motivation and the chosen teaching education program. There is a comparatively high proportion of extrinsically motivated students among elementary school student teachers, while a high proportion of intrinsically motivated students predominate among secondary school student teachers. This result complements the studies by Retelsdorf and Möller (2012) and Grüneberg and Süß (2023) on a subject-specific level.

Other studies also deal with the subject-specific motivation of university students. Fischer and Bisterfeld (2015) compared the motivation of sport students for choosing a degree in teacher education (subject-specific) with the motivation of student teachers in general (non-subject-specific). The analysis of the findings reveals that the intrinsic motivation factors with educational interest, ability beliefs, and subject-specific interest are more prevalent than the extrinsic ones. Overall, the subject-specific comparison shows only minor differences: personal career-related ability beliefs and subject-specific interest are less relevant for sport students than for student teachers of other subjects (Fischer and Bisterfeld, 2015).

Fischer and Golenia (2021) also investigated the question of why physical education students decide to choose teacher education as a study program. The four profiles identified each relate to a characteristic bundle of motivations and show that sport students are a heterogeneous group in terms of their decision-making motivation. This study (Fischer and Golenia, 2021) complements previous findings that have identified the intrinsic pedagogical motivation of sport students and student teachers in general as the main decision-making motivation.

### 2.2 Studying music

An analogy between sport and music exists due to the extensive biographical experiences in both areas, which has already been established in many contexts, e.g., in the context of biographical work (Immerz and Tralle, 2022). It can therefore be assumed that childhood and adolescent experiences form a kind of “background foil” [Hintergrundfolie] (Volkmann, 2008, p. 14) for music students as well as sport students even before they begin their studies.

In her biographical study of the lifespan development of professional musicians, Manturzewska (2006) describes six phases with typical developmental tasks, the first three of which are particularly relevant in the context of “music studies.” The development of sensory, emotional, and aesthetic sensitivity to music begins from birth to age 5 (phase 1). The intensive development of musical skills on the instrument (phase 2: 6–14 years) is followed by the development of the artist’s personality and self-confidence as an artist (phase 3: 15–25 years). This phase usually includes the decision to study and the choice of subject and study program. Against this background, Nagel (1988) has pointed out that early specialization in one instrument is desirable for technical and artistic proficiency and necessary for becoming “a serious musician” (Nagel, 1988, p. 74). However, this early specialization and focus on a career in music could also limit and narrow opportunities to explore non-musical alternatives. In most education systems, the first important phase of musical development ends with the school-leaving certificate at the transition from adolescence to early adulthood and finally leads to the vocational orientation phase (Spahn, 2015).

This transition between school and university or university of music has been examined in some studies from the students’ perspective. Váradi et al. (2024) interviewed students in the final year of a vocational secondary school with music specialization about their career choice and professional motivation. They found that the main subject teacher – the instrument teacher – plays a decisive role and often influences the personal path of the students and the professional career. The immediate family environment, social ties, belonging to the community, and the idealization of the profession also proved to be possible motivations for a career in music. Guan et al. (2022) also investigated the mechanism between the school music context and the music career choice of young people in secondary school. They found that school music context significantly predicted the choice of a career in music. Furthermore, music motivational beliefs (i.e., music value and music interest) served as a mediator during this path and partially explained the psychological process between school music context and music career choice. Rickels et al. (2019) carried out a comparative analysis of influences on choosing a music teaching occupation. Their purpose was to compare motivations and influences of high school music students who express an interest in a career in music and those who do not. They compared three occupation groups using a discriminant analysis of the resulting components and found differences between the groups pursuing music teaching, other music, and other non-music. Four components were identified that could be considered as potential archetypes in future research considering selectors: full immersion musician/leader, full immersion teachers, certain aspirational leader, family, and non-leader. The authors conclude that career choices (to pursue music teaching or other choices) are more multidimensional than accounted for in previous studies (Rickels et al., 2019).

Other studies examined the perspective of music university students. Wang and Wong (2022) investigated a comprehensive cognitive motivation model and were able to prove that intrinsic motivation is an effective determinant of career intentions and decisions. The results show that cognitive factors, motivation, and environmental factors have different degrees of influence on students’ professional intentions and together influence students’ career intentions. Miksza et al. (2021) tested a theoretical model of a network of relationships among perceptions of competitiveness, perfectionism, teacher control, quality of motivation, and intentions to pursue a career in music. They found that the quality of motivation appears to be a good explanation for career endorsement, with intrinsic motivation strongly associated with higher intentions to pursue a music career. Path analyses showed that those with stronger career intentions also have stronger intrinsic motivation orientations. In their study, Von Georgi and Lothwesen (2010) investigated the question of whether music students at educational institutions with an artistic focus have increased competence and motivation to perform compared to students at other universities. Results show that the two scales of motivation and empathy can be interpreted as basic motivational factors for behavior and study. The dimension of motivation to study is formed by a fundamental attitude toward the general importance of music in personal experience, whereby music as a study interest moves strongly into the center of the motivational explanatory approach. Parkes and Jones (2012) investigated motivational constructs influencing undergraduate students’ choices to become classroom music teachers or music performers. Using stepwise multiple regression, the authors documented that attainment value, intrinsic interest value, and expectancy predicted 74% of the variance in whether students intended to choose a career teaching music. They found that expectancy, attainment value, ability perceptions, and intrinsic interest value explained 65% of the variance in whether students intended to choose a career in music performance.

While Parkes and Jones (2012) compare student music teachers with music performers, other studies refer only to the teaching profession with music as a subject. Neuhaus (2007) deals with the career choice process of student education teachers in music and found that music studies were chosen primarily out of an interest in the subject of music and the desire to become a teacher at public school. However, it did not play a role as an opportunity or alternative course of study; rather, the students consciously chose the study program. Weiß and Kiel (2010) also found that prospective music teachers to some extent rely on individual interests, expectations and associated professional demands when they decide to start a study as a teacher of music. Thereby, the subject-motivation of prospective teachers of music as a whole does not differ from that of other student teachers, but Weiß and Kiel (2010) found a reduced importance of the integration of work and family for the students of music. According to Thornton and Bergee’s (2008) study another finding can be noted: 56% of the participants stated that significant others, especially music teachers, who were involved in their lives had an influence on their decision to study music.

### 2.3 Specifics of music and sport studies

Music and sport are among the degree courses for which an entrance examination is required. As this can influence the motivation to study in the context of the present study, differences between music and sport will be briefly outlined here.

In the field of music, applicants for a bachelor degree (Bachelor of Music – B.Mus.) prepare mainly for an artistic examination in *one* main instrument, for example, violin, trumpet, or voice. In addition to this artistic focus, they are examined in the compulsory subject of piano as well as in the areas of music theory and ear training. The entrance examination for teaching education with music as a main subject is broader in scope. Beside their main instrument, applicants are tested in piano and singing as well as in music theory and ear training. They are also asked about their motivation and their pedagogical skills for studying to become a secondary school teacher.

In the field of sport, there is usually a joint examination for those interested in either the Sports Science degree program (Bachelor of Science – B.Sc.) or the teacher education program with sport as a teaching subject. Applicants are tested in five different sporting disciplines: athletics, swimming, apparatus gymnastics, games, and gymnastics. In each discipline, two to four sub-areas are tested. For example, basketball, handball, soccer, and volleyball are required in the discipline *games*. The whole examination lasts a full day.

In a comparison of the two areas of music and sport, it can be stated that the entrance examination in music is more specialized and focuses on musical-artistic performance in one or two main instruments, while in sport, many sub-disciplines must be equally mastered.

**Figure 1:**
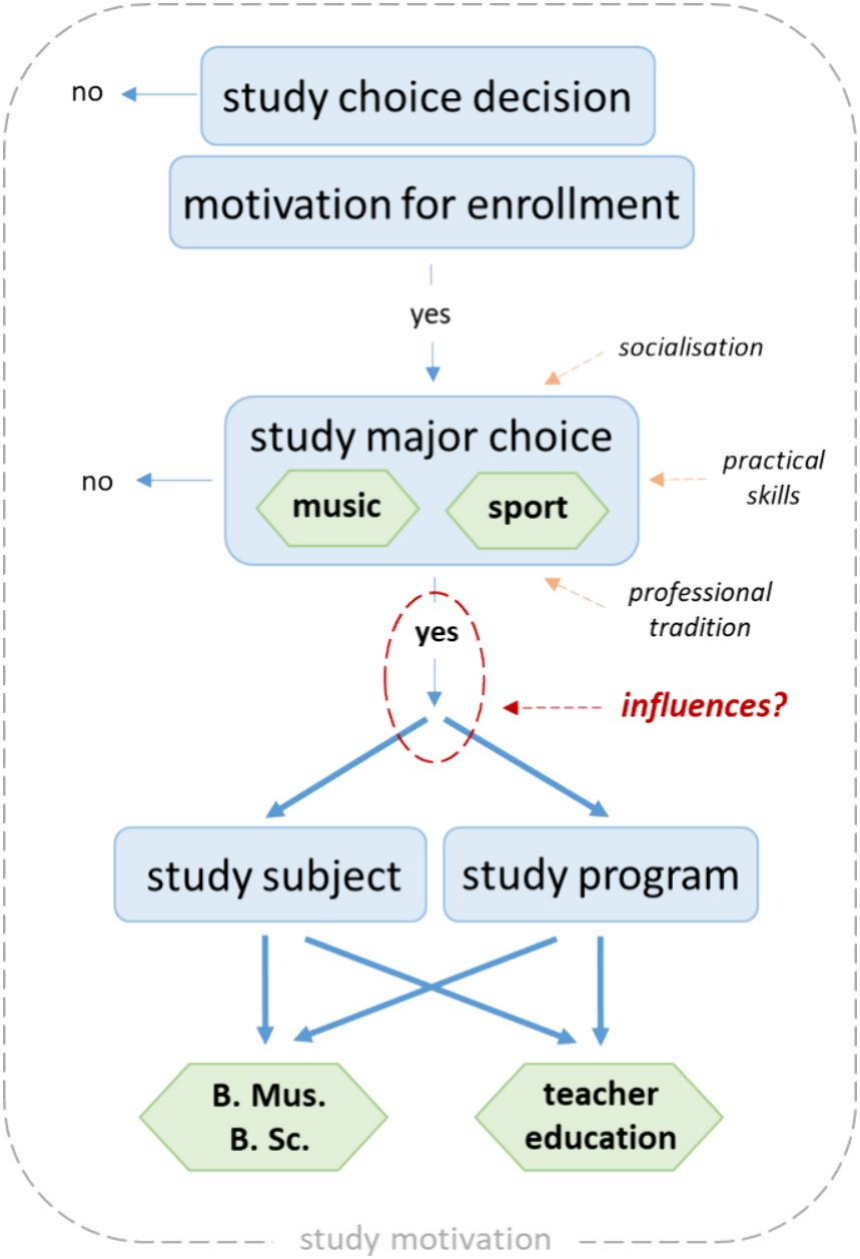
Students’ path to study music and sport.

Figure 1 outlines the decision-making process and possible pathway for students to study music or sport, based on the literature described above. This graphic illustrates the complex issues surrounding the topic of students’ study motivation, also taking into account the educational biography, the identity formation process of the students and their social background. The following research questions focus on the area marked in red.

In summary of the previous literature it becomes clear that music and sport are supposedly special subjects that have received relatively little attention in previous research on study motivation from the university students’ perspective. Although there are comparisons between music and sport in other studies, the samples are quite different, for example in a recent study by Hatfield (2024) on determinants of motivation in world-class musicians and Olympic athletes, which relates to the continuation or termination of a successful career. Nevertheless, striking and contrasting motivational patterns were identified throughout the development of the six artists interviewed, which are also applicable to the question for study motivation in our investigation. It was found that the artists who continued their careers were largely driven by autonomous forms of motivation such as self-initiative, passion, curiosity, and a desire for endless discovery and aspiration. In contrast, controlled coercive forms of motivation drove the artists who gave up their careers.

Other studies refer only superficially to the subjects of music and sport or point to a loose connection between the subjects. Kaub et al. (2016), for example, included the subject combination of art and music in their study, but subsumed it under the group of humanities scholars and linguists. In contrast, Glutsch et al. (2018) found that the subjects of sport, music, and art could not be assigned to any subject group that can be studied at German universities. The special feature and parallelism of the subjects is that they are probably associated with a “special talent” (Glutsch et al., 2018, p. 466). Against this background, Kaub (2015) poses the question of whether the subjects of music and sport are associated with more enthusiasm, interest or professional motivation than other subjects. This question leads to the research gap that this study aims to address.

### 2.4 Present study: aims and research questions

The aim of the present study is to investigate university students’ motivation for their decision to pursue their course of study, their motivation of enrollment, and their study major choice in music and sport. It also aims to identify similarities and differences between the subjects of music and sport. There are three main research questions:

1. What are students’ study motivations for pursuing music and sport studies?
2. Are there similarities and differences of study motivation between music and sport, and if so, what are they?
3. Are there differences in the students’ motivation to study music and sport compared to other subjects?

## 3 Methods

### 3.1 Study design

The study sample consisted of university students studying music at the University of Music Freiburg or sport at the University of Freiburg. Students at the beginning of their studies were asked about their motives and reasons for choosing their study subject or degree program.

The study was performed as an online survey^1^. At the University of Music Freiburg, the survey was distributed and carried out in on-campus lectures. At the end of the lectures, an experimenter explained the study procedure and information on data protection and provided the link to the study on screen. The sports university students were given the link by e-mail via the Head of the Chair of Sports Psychology at the Institute of Sport and Sports Science at the University of Freiburg. The mail also contained information about the study and data protection.

On the first online page, the students had to give their consent to participate in the survey. The ethics committee of the Freiburg University of Music gave ethical approval for the conduct of this study.

### 3.2 Participants

In this study, a total of 151 university students participated. The sample included 110 music university students and 41 sport university students (Table 1). There were 51% female, 48% male and 1% diverse students without statistical distribution difference between music and sport university students. 70% of the students were in the first year of study and 30% in the second year and higher with no difference in distribution between the study subjects. The mean age of the participants studying music in the first year of study was 19.9 years (SD = 2.0 years, *N* = 79). The mean age of first-year university students in sport was 20.9 years (SD = 2.2 years, *N* = 26) with significant distribution difference between music and sport (*p* = 0.024). For the study program, 57% were in the Bachelor of Music (B.Mus.) and Bachelor of Science (B.Sc.) study programs and 43% studied for secondary school teacher education in music and sport. The mean age when the university students started with instrumental practice or sport training was 6.5 years (SD = 2.7 years) without significant difference between the study subjects.

**Table 1:** Sample characteristics.

	Music university students	Sport university students
Amount	*N* = 110 (73%)	*N* = 41 (27%)
Gender	49% female, 50% male, 1% diverse	57% female, 43% male
Semester	95% first year of study 28% second year or higher (with 74% 3. semester)	81% first year of study 37% second year or higher (with 47% 3. + 4. semester)
Mean age (SD) of first-year-students	19.9 years (2.0 years) *N* = 79	20.9 years (2.2 years) *N* = 26
Study program	62% Bachelor of Music (B.Mus.) 38% Teacher Education *Music*	44% Bachelor of Science (B.Sc.) 56% Teacher Education *Sport*
Mean age (SD) of start music/sport	6.6 years (2.7 years)	6.0 years (2.6 years)

### 3.3 Questionnaires

The study contained a custom-made questionnaire on sociodemographic data and two standardized questionnaires.

The questionnaire on socio-demographic data consisted of questions on age and gender, and questions about the study, i.e., the study program, the main instrument or sports area, and the semester of study. In addition, questions about biographical musical or sports background were included. For that, the students were asked at what age they started actively making music or practicing sport (see Table 1). They were also asked about their age when the decision for studying music or sport was made.

Furthermore, a question was added that relates to an alternative to the choice of this study. The item “Had you considered an alternative to studying music/sports?” was answered on a four-point scale with 1 (“no”) to 4 (“yes”).

#### 3.3.1 Questionnaire on motivation for enrollment (STUWA)

The STUWA questionnaire used is a multi-factorial inventory to measure motivation for enrollment (STUWA: Ein multifaktorielles Inventar zur Erfassung von Studienwahlmotivation; Janke et al., 2023). Five of the scales were selected for the survey: (1) intrinsic, (2) extrinsic-materialistic, (3) extrinsic-social, (4) socially induced study motivation and (5) uncertainty of the study choice (see Table 2). Each of these subscales contains three items. The scale ranges from 1 (“not true at all”) to 7 (“completely true”).

**Table 2:** Mean values of the STUWA scales by study subject and program.

STUWA scales	Study subject	Study program	Mean	SD	*N*	Statistics
Intrinsic (*M* = 5.98)	Music	B.Mus.	6.29	0.78	65	*p* = 0.001
		Teacher Education	6.19	0.64	42	*p* = 0.021
	Sport	B.Sc.	6.19	0.51	17	*p* = 0.051
		Teacher Education	5.97	0.68	23	n.s.
		Total	6.20	0.70	147	n.s.
Extrinsic-materialistic (*M* = 4.98)	Music	B.Mus.	3.35	1.58	65	*p* < 0.001
		Teacher Education	4.61	1.39	42	*p* = 0.047
	Sport	B.Sc.	3.47	1.11	17	*p* < 0.001
		Teacher Education	5.11	1.21	23	n.s.
		Total	4.00	1.59	147	*F*(3,143) = 12.4, *p* < 0.001
Extrinsic-social (*M* = 4.48)	Music	B.Mus.	4.22	1.48	65	n.s.
		Teacher Education	5.07	1.48	42	*p* = 0.007
	Sport	B.Sc.	4.60	1.19	17	n.s.
		Teacher Education	5.85	1.20	23	*p* < 0.001
		Total	4.76	1.52	147	*F*(3,143) = 8.5, *p* < 0.001
Socially induced (*M* = 3.01)	Music	B.Mus.	3.49	1.63	65	*p* = 0.010
		Teacher Education	3.87	1.52	42	*p* < 0.001
	Sport	B.Sc.	3.00	1.25	17	n.s.
		Teacher Education	3.98	1.30	23	*p* < 0.001
		Total	3.62	1.53	147	n.s.
Uncertain (*M* = 3.23)	Music	B.Mus.	2.40	1.45	65	*p* < 0.001
		Teacher Education	3.28	1.48	42	n.s.
	Sport	B.Sc.	3.70	1.42	17	n.s.
		Teacher Education	2.81	1.34	23	n.s.
		Total	2.86	1.50	147	*F*(3,143) = 5.4, *p* = 0.002

[SD, standard deviation; significances show the *t*-tests compared to the mean scale value of the general student population (in the first column in brackets), and statistics of the MANOVA in each scale across the study subject and program].

(1) The *intrinsic* motivation scale includes items that relate to the content of the subject. University students assess whether they enjoy studying the content of the subject and find it interesting. They also assess whether the subject matches their abilities and talents. (2) The *extrinsic-materialistic* scale relates to financial aspects. University students assess whether they have chosen their studies in order to earn well, to be financially secure and to have a secure income later on. (3) The *extrinsic-social* scale focuses on the compatibility of work and family, friends, and hobbies. For example, university students are asked whether they chose their degree course in order to be able to look after their family alongside their career. (4) The *socially induced* scale refers to relevant other people who encouraged the university students to choose their course of study. The items ask about friends, family and people with whom the students have worked and who think they should choose their study degree program. (5) In addition to more established facets of study motivation, the STUWA also makes it possible to record the degree of *uncertainty* of the study choice. Students choose their degree course because they are not sure what career they would like to pursue later, they do not know exactly which degree course suits them, or they are generally unsure which degree course is right for them. For each scale, the mean scale values of a general student population taken from Janke et al. (2023) were used for a comparative analysis.

#### 3.3.2 Aspects of importance in the profession

The students were asked to what extent certain aspects of their future profession are important to them. With the question “What is important to you for your future profession?” six aspects had to be rated on a four-point scale from 1 (“not important”) to 4 (“very important”). These aspects were: personal interest, good job prospects, good earning opportunities, career opportunities, social reputation, and the continuation of an already started education. This range of aspects was also used in other student surveys, and the results of some aspects in the general student survey 2022 (Hinz, 2022) were used for comparison.

### 3.4 Statistics

The analyses were carried out with SPSS 29 (Armonk, NY: IBM Corp). Descriptive statistics include the mean value and the standard deviation (SD) of the mean. Nonparametric comparisons were examined using cross tables reporting Pearson’s *χ*^2^. *T*-Tests (single-sided) were used between the mean scale value and the value of the scale in the general student population. Multivariate analysis of variance (MANOVA) was used for the comparative analyses with all STUWA scales between the study subjects and program. On significance, *post hoc* analysis was performed using Tukey-HSD correction. The level of significance was set to *p* = 0.05.

## 4 Results

### 4.1 Motivation for enrollment (STUWA)

The mean values of the STUWA scales by study subject and study program are listed in Table 2.

The results showed no significant difference among university students in the scale *intrinsic* motivation to study. However, the music students had significantly higher mean values compared to the mean value of this scale in the general student population.

There was a significant main effect in the mean values among university students for *extrinsic-materialistic* motivation to study, particularly between students in the bachelor and those in the teacher education program. Bachelor students in both study subjects had significantly lower scores than the teacher education students (*post-hoc*, *p* < 0.032) and compared to the general student population. The student teachers tended to show similar values to the general scale value.

In the *extrinsic-social* motivation scale, the significant main effect between the university students was similar to the extrinsic-materialistic scale especially caused by the differences between the bachelor and teacher education students (*post-hoc*, *p* < 0.033). While the bachelor students had a lower but similar mean value than the general student population, the teacher education students showed significantly higher values.

The mean values in the *socially induced* scale did not differ among the university students. However, with the exception of the sports students in the Bachelor of Science program, the values were significantly higher than in the general student population.

There was a significant main effect among university students in the scale of *uncertain* choice of study, which was mainly caused by the low mean value of the music students in the Bachelor of Music program (*post-hoc*, *p* < 0.015).

### 4.2 Biographical background and professional importance

**Figure 2:**
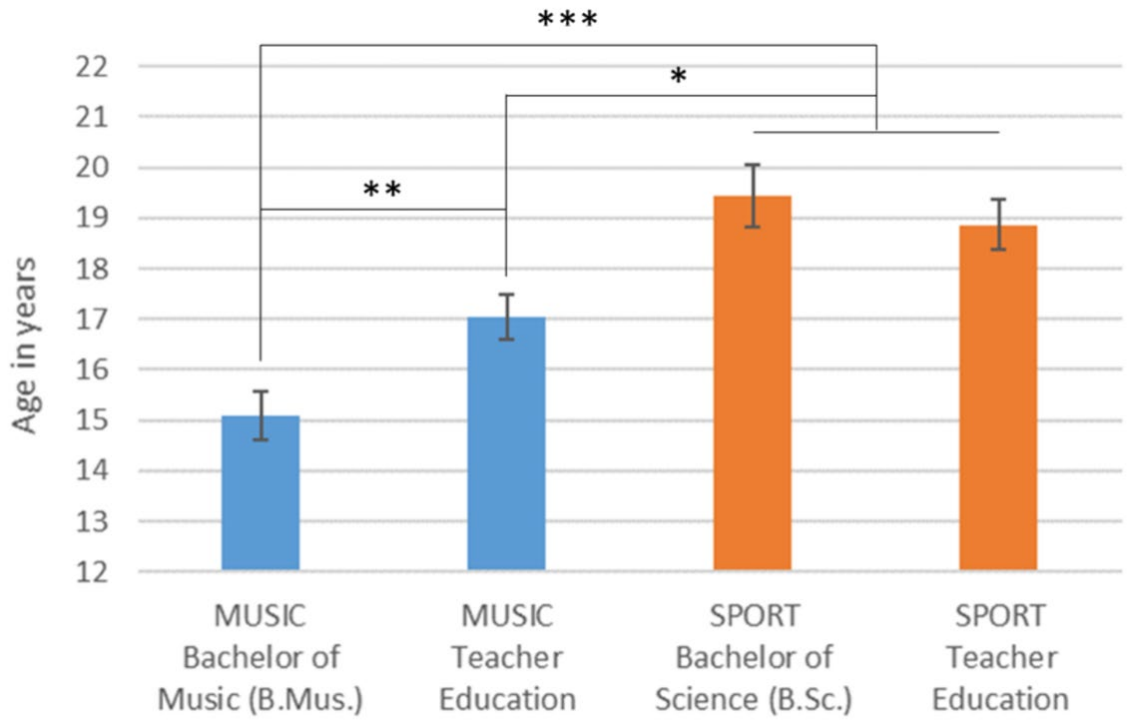
Mean age of deciding to study music or sport by study subject and program (error bars: standard error of the mean. *p < 0.05; **p < 0.02; ***p < 0.01).

The average age at which the decision for studying music or sport was made differed significantly among the university students [Figure 2; *F*(3,147) = 12.5, *p* < 0.001]. The music university students in the Bachelor of Music program were the earliest to make this decision (*post-hoc*, *p* < 0.013).

**Figure 3:**
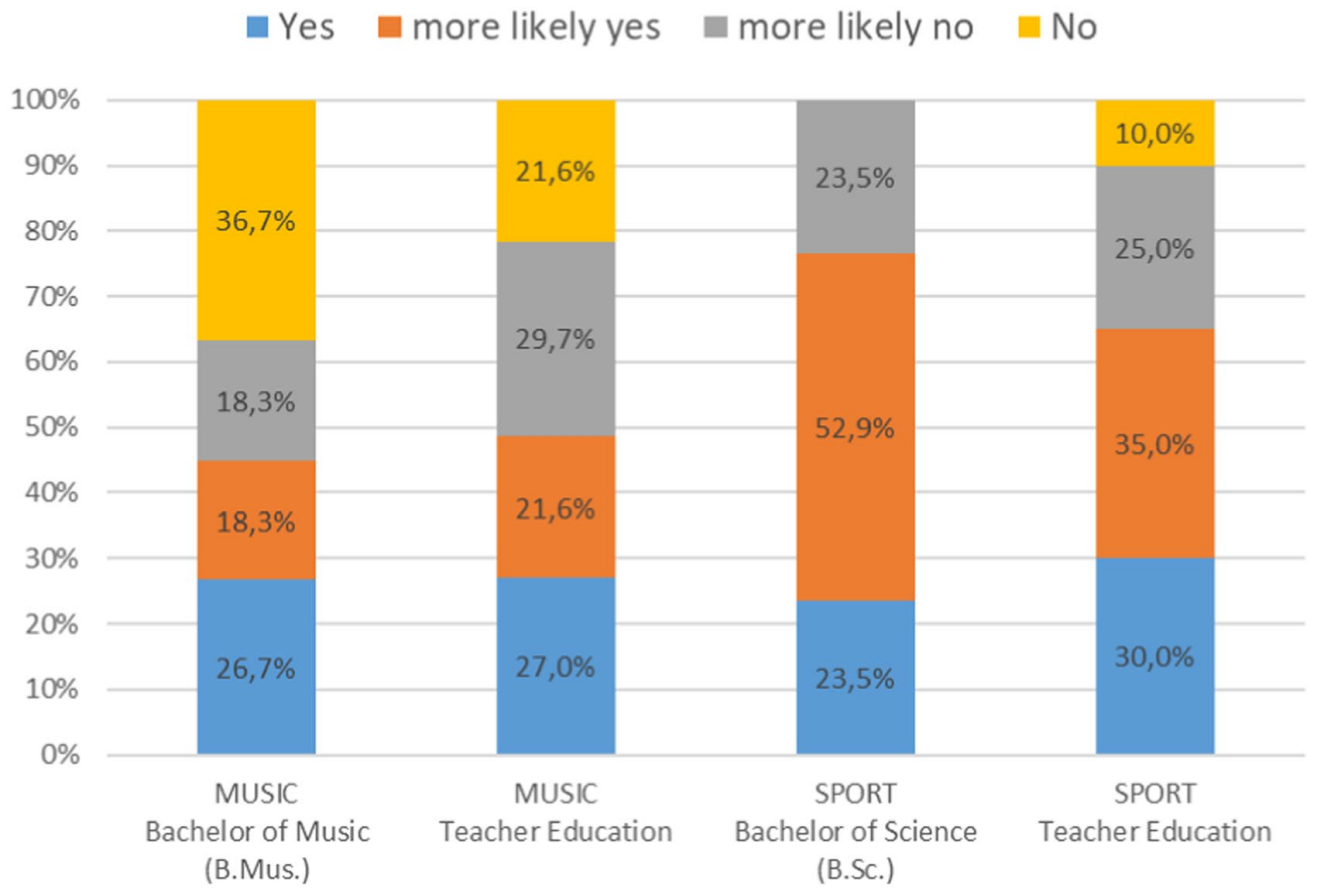
Histogram of the answers to the question if the university students had an alternative to study music or sport.

With regard to the question of whether the students had an alternative to study music or sport, there was a significant difference in the distribution of responses (Figure 3; Chi^2^ = 18.3, *p* = 0.032). The music students in the Bachelor of Music program had less of an alternative in mind than the sport students in the Bachelor of Science program.

**Figure 4:**
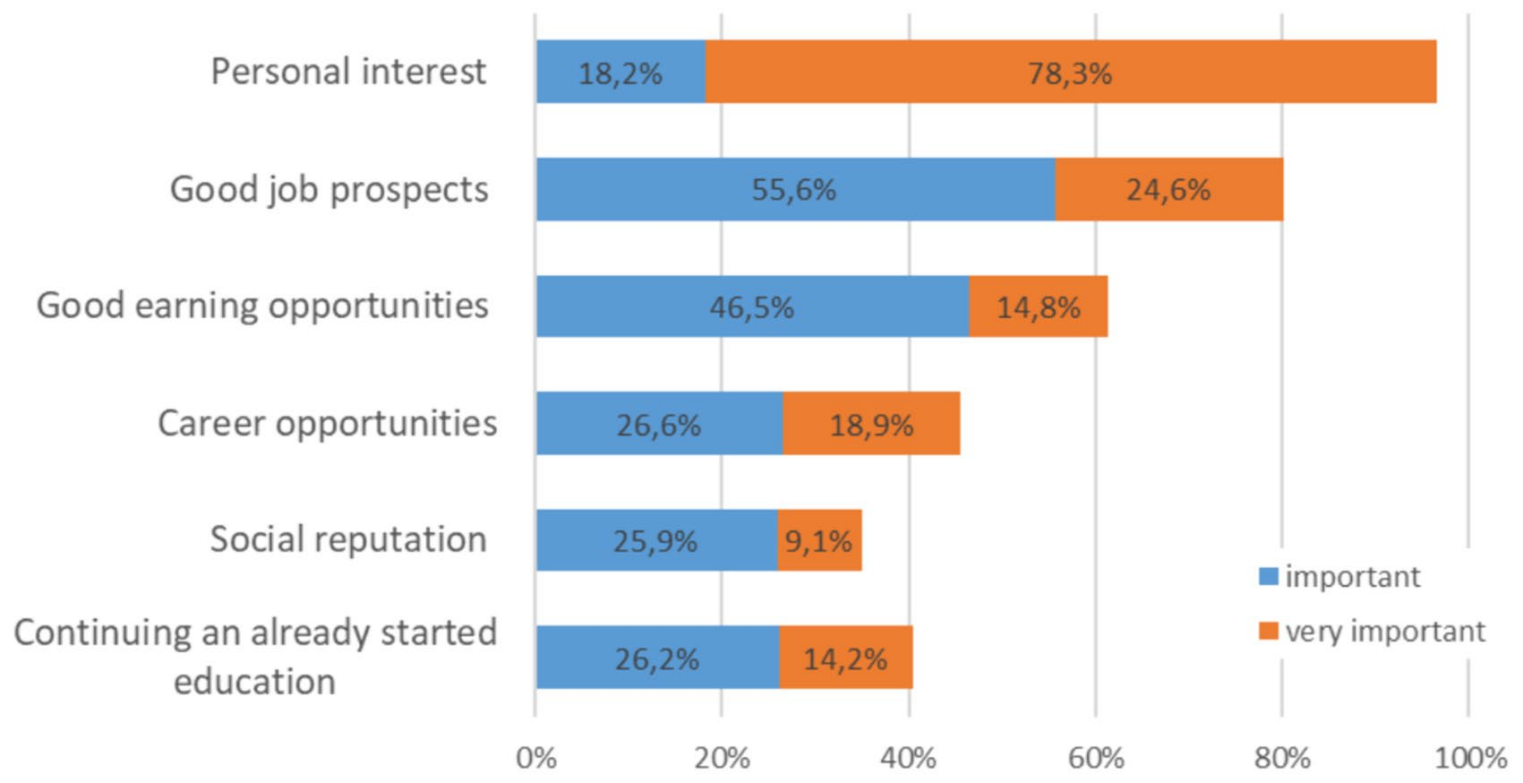
Distribution of the answers to specific job-related aspects across all students to the question, What is important for the future profession?

When asked what is important to the students for their future profession, the students answered rather similarly, regardless of the study subject and study program (Figure 4). Most of all, personal interest was rated highest of all with 78% being very important. Regarding the good earning opportunities, the music and sports students rated this aspect less important, with 14%. The only significant distribution differences in these job aspects between the university students in music and sport were found for career opportunities [Chi^2^ = 37.2, p < 0.001] and in continuing an already started education [Chi^2^ = 19.5, *p* = 0.021]. The music students in the Bachelor of Music program and the sport students in the Bachelor of Science program rated career opportunities as more important (important to very important >60%) than the teacher education students (about 20%). The continuation of an already started education was most important for the music students in the Bachelor of Music program (important to very important 56%) in comparison with the other university students (<30%).

## 5 Discussion

In this project, the study motivation of music and sport students at their beginning of their study at the University of Freiburg and the University of Music Freiburg was examined. The results showed that there are many similarities between music and sport university students, but also differences with regard to the study programs within the subjects.

### 5.1 Students’ study motivation

Both music and sport university students had a similarly high intrinsic study motivation. The level of intrinsic motivation seems to underline their passion and commitment to their respective field. This result is in line with the findings of Bornhorst et al. (2020) on high study motivation over all university students with a high level of commitment to the subject. It was found in our study that the intrinsic motivation of sports students was similar to that of the general student population whereas that of the music students was higher. Other studies have also shown high levels of intrinsic motivation in sport students (Fischer and Bisterfeld, 2015; Fischer and Golenia, 2021), which is particularly pronounced in teacher training students for secondary school (Retelsdorf and Möller, 2012; Streblow and Brandhorst, 2018; Grüneberg and Süß, 2023) and corresponds therefore partly to the sample in this study. Studies among music university students show that intrinsic motivation is an effective determinant of career intentions and decisions (Wang and Wong, 2022; Hatfield, 2024) and is strongly associated with higher intentions to pursue a music career (Miksza et al., 2021), which could explain the higher intrinsic motivation.

In terms of extrinsic-materialistic motivation, a difference is particularly evident between the different study programs. Thus, materialistic goals motivated the teacher training students more than the bachelor students in the artistic-oriented study program. In this case, the student teachers seem to correspond to type 3, according to Bornhorst et al. (2020), who increasingly cite external study motivations such as a good income and a permanent position. Nevertheless, these teacher training students differ greatly from other groups, such as business students, who choose their studies out of an extrinsic-materialistic motivation to a much greater extent than students of other subjects (Janke et al., 2023). On the other hand, it is interesting to note that a lucrative future played less a role for the bachelor students in music and sport equally. This finding can possibly be explained by the prospects of the two future professions. Despite the different requirements in the subjects of music and sport, the teaching profession is a clearly defined field of activity with a manageable structure, secure career prospects and a regular income. This result presumably reflects the desire of student teachers for financial security. However, the professional field and area of activity following a bachelor degree in music and sport is less clearly defined compared to the teaching profession. It can only be assumed that the students are aware of this uncertainty, have nevertheless decided to study music and sport, and are open to various career prospects. It can be stated that the differentiation of materialistic goals between teacher training students and bachelor students provides important insights into the different priorities and professional expectations within the two subjects.

The difference between study programs was also found for the extrinsic-social scale where the teacher training students answered with higher scores regarding the wish to support a private and a family life with the future profession. This is consistent with the results of Grüneberg and Süß (2023) and Janke et al. (2023), in which student teachers stated that the compatibility of family and career was important to them and that the teaching profession offered good employment opportunities, flexibility, free time, and compatibility with family life. Weiß and Kiel (2010) found that the integration of work and family was less important for student teachers of music, but this is not reflected in our data as a subject-specific difference between music and sport and students of other subjects. It seems that the teacher students like to see a secure future and regard the teaching profession as such, where the bachelor students did not foresee the upcoming profession and initially the study itself was the main and most important motivational target.

With regard to socially induced study motivation, it was shown that music students and sports teacher training students were more socially induced to study their subject compared to the general student population. This high socially induced study motivation was also reported by student teachers in other studies (Pohlmann and Möller, 2010; Janke et al., 2023), which means that these students decided to study because they were encouraged by friends, family, or colleagues. This seems to be an important result, especially for music-related studies, as it is known from other studies that instrumental teachers and the family environment (Váradi et al., 2024), the school music context in secondary school (Guan et al., 2022), or music teachers as relevant others (Thornton and Bergee, 2008) can influence the decision to study music. In addition to the musical and sporting basics and skills that must be learned for the two subjects, these relevant others also seem to be decisive in the biographical path of students and ultimately their choice of study.

In this regard, the scale uncertainty of the study choice showed that the Bachelor of Music students were less uncertain that they wanted to study exactly this subject than the other students of the sample. However, student education teachers in music indicate more uncertainty with regard to their choice of study compared to student teachers of other subjects (Janke et al., 2023). It suggests that this is not a music-specific phenomenon, but could be a special characteristic of the Bachelor of Music degree program. In terms of data, these students choose their degree course because they are sure that they want to make music – highly intrinsically motivated – and want to specialize in their instrument or with their voice, and because they know that this study program suits them and is right for them. Both the differences in extrinsic-social motivation and certainty in course choice, provide insight into how social contexts and prior experiences influence students’ academic decisions.

### 5.2 Biographical background in music or sport and importance in the profession

Based on the literature, a wide range of biographical experiences (Volkmann, 2008; Lüsebrink et al., 2014; Bornhorst et al., 2020; Immerz and Tralle, 2022) and an early and intensive involvement with music (Nagel, 1988; Manturzewska, 2006; Spahn, 2015) and sport (Klinge, 2002; Miethling, 2018; Wylleman, 2019; Wyllemann et al., 2020) can be identified as similarities between both areas. These aspects can also be found in the data of our study.

On the one hand, it was shown that both groups – music and sport students – began their activities with instrumental practice or sport training at around the same time, at an average age of 6.5 years. Thus, it can be assumed that these activities are related to the school age in the German school system and that the transition from kindergarten and entry into primary school is therefore framed by extracurricular activities such as instrumental lessons or joining a sports club. On the other hand, there is an interesting difference in terms of age and entry to university or university of music. Music university students in their first year of study are on average 1 year younger than sports university students. However, they were not only younger when they started their studies, but also decided to study music much earlier than the sports students did. What music and sport have in common is that many years of previous experience and intensive preparation are necessary in order to be able to make the decision to study music or sport in order to get a place at university or university of music. The “staircase” in Figure 2 indicates that a high degree of specialization is taking place in music, which is reflected in the early decision to study. This is probably also related to the format of the entrance exam in both subjects. While an enormous range is tested in sport for Bachelor of Science and teacher training, the examination format for a teacher education degree in music focuses on far fewer areas. The specialization on one main instrument becomes very clear in relation to the Bachelor of Music degree course. These findings that music students show less uncertainty in their choice of major and make this decision at a younger age compared to sport students enrich the understanding of the academic cultures of music and sport, but also suggest potential areas for future comparative studies with other study programs and disciplines.

In the question of whether the university students had an alternative to study music or sport, it was found that the music students in the Bachelor of Music had fewer alternatives in mind than the sport students in the Bachelor of Science. Following Nagel’s (1988) argument, an early specialization in one instrument and an early focus on a career in music can also limit non-musical alternatives in the study choice. This is also reflected in this data for Bachelor of Music students in their early decision to study. Based on the data available, it is not possible to answer whether the students have decided on a profession for which they are aiming with their studies or have merely decided to study and are open to alternative career options (Neuhaus, 2007; Parkes and Jones, 2012; Rickels et al., 2019). When asked about study alternatives, the results for student education teachers in music and sport differ from those in other subjects. For teacher training students of all subjects, nearly 88% said that teacher education is the preferred choice of study (Grüneberg and Süß, 2023) and also in music, students consciously chose music as a teaching subject (Neuhaus, 2007). The data of this study is less homogeneous. While 51% of students in the music teaching degree stated that they had more likely no or even no alternative, for teaching education in sport this figure was 35%. In contrast, there are almost 49% of university students with an alternative in music and as many as 65% of university students with an alternative in sport. One explanation here could also be the much broader focus in sport compared to the focus on one main instrument in music. However, for further statements as to whether these are subject-specific characteristics, further studies would have to be carried out.

Among the students’ answers as to what is important for their future profession, there is no difference concerning the study subject or study program. Most of all, personal interest was rated highest of all with 78% being very important. This was very similar to the answers of other students studying humanities (78%) and cultural sciences (75%) but different to law students (38%) and business science students (35%) (Hinz, 2022). Regarding the good earning opportunities, the music and sports students rated this aspect less important, similar again with the students of cultural (15%) and literature sciences (18%), and in contrast to the law (51%) and engineering/computer science students (48%). This can also be seen in other studies with the STUWA (Janke et al., 2023), in which business students choose their studies out of extrinsic-materialistic motivation with the aim of earning well later on, being financially secure, and having a good income.

### 5.3 Limitation and future research

The limitations of the study are mainly due to the relatively small sample size. Thus, the results and implications can only be generalized with reserve, especially as this was a group from one University of Music and one University.

There are numerous starting points for further studies. It would be interesting to differentiate more precisely between certain areas such as teaching education and bachelor degrees and possibly the academic and family background of university students in music and sport. The question of whether the subjects of music and sport are associated with more enthusiasm, interest, or professional motivation than other subjects (Kaub, 2015) cannot be answered conclusively on the basis of the present data. However, there are indications that this is the case for music, especially for students on artistic degree courses. In this regard, an in-depth investigation of the study motivation of traditional and non-traditional students (Brändle, 2014) in music-related degree programs could also be interesting.

Within the subject of music, future research on students’ motivation to study in relation to different musical specialties would certainly offer potential. This is particularly interesting in light of the changing musical landscape, the emergence of new professional fields and the increase in musicians’ portfolio careers. A comparison with other subjects would also be desirable. For this purpose, the STUWA questionnaire (Janke et al., 2023), as inventory to measure study motivation, should also be used in other subjects and study programs in order to obtain comparative material. In addition to the university students who have decided to study music or sport, there are many teenagers and young adults who are intensively involved in music or sport and are very interested in these areas, but do not decide to study these subjects. It would be interesting to include this group in further studies in order to understand the reasons why young people choose another subject of study over music or sport.

## 6 Conclusion

In the present study, students’ motivation to study music and sport was examined and for the first time compared between the subjects and of different study programs within the subjects. The engagement with the subject content – learning an instrument or training in a sports club – begins early in childhood for students in both subjects. Another similarity is that music and sports students are equally or more intrinsically motivated than students in other subjects. What is particularly interesting, however, is that there are differences in the subjects depending on the study program, as the bachelor students in music (Bachelor of Music) and sport (Bachelor of Science) differ from the teacher education students in music and sport. Thus, extrinsic-materialistic and extrinsic-social motivation is higher among teacher training students than bachelor students. There is a subject-specific difference in the younger age of first-year-students in music, the earlier decision to study music and the fewer study alternatives of bachelor of music students. In addition to other studies on students’ motivation for study choice decision, their motivation of enrollment and their study major choice, the focus on the subjects of music and sport and the comparison provides interesting insights into these subject cultures.

## Data availability statement

The raw data supporting the conclusions of this article will be made available by the authors, without undue reservation.

## Ethics statement

The study has received a positive vote from the ethics committee of the University of Music Freiburg. Consent to participate in this study was provided by the participants.

## Author contributions

AI: Conceptualization, Project administration, Writing – original draft, Writing – review & editing. MN: Conceptualization, Data curation, Formal Analysis, Project administration, Methodology, Writing – original draft, Writing – review & editing. JH: Conceptualization, Methodology, Writing – review & editing. CS: Conceptualization, Writing – review & editing, Supervision.

## Footnotes

^1^ soscisurvey.de
